# Abdominal actinomycosis in an elderly diabetic

**DOI:** 10.12669/pjms.36.ICON-Suppl.1724

**Published:** 2020-01

**Authors:** Riyasat Ahmed Memon, Yusra Shafquat, Nausheen Yaqoob

**Affiliations:** 1 Riyasat Ahmed Memon, MBBS. Registrar, Section of Histopathology, Department of Pathology, The Indus Hospital, Karachi, Pakistan; 2 Yusra Shafquat, MBBS, FCPS. Consultant Microbiologist, Section of Microbiology, Diagnostic and Research Laboratory, Liaquat University of Medical and Health Sciences, Hyderabad, Pakistan. The Indus Hospital, Karachi, Pakistan; 3 Nausheen Yaqoob, MBBS, FCPS. Consultant and Head of Section of Histopathology, The Indus Hospital, Karachi, Pakistan

**Keywords:** Actinomycosis, Elderly Diabetics

## Abstract

Actinomycosis is a rare infectious disease that presents as three entities, cervico-facial, abdominal and genital, with cervico-facial being the commonest. Due to its subacute presentation and indolent course, abdominal actinomycosis is difficult to diagnose and is often confused with malignancy. We present a case of an elderly diabetic with no known other risk factors of the disease with complaints of right sided abdominal swelling and presence of abdominal mass on imaging, diagnosed post operatively as a case of abdominal actinomycosis, on histopathology. Abdominal actinomycosis should be considered in differentials in cases with abdominal masses. Diabetes Mellitus is not an established risk factor for development of abdominal actinomycosis. Studies are required to link its association with the disease.

## INTRODUCTION

Actinomycosis is a rare, progressive infiltrating disease that is caused by *Actinomyces israelli*, a gram positive microaerophilic organism that is part of normal oral, gastrointestinal tract and genital tract flora.^1^-^5^ It is known to be non-pathogenic, causing disease in patients with a breach in mucosa or those with impaired immune function.^1^,^5^ It is known to have three entities – cervico-facial, abdominal and genital, with cervico-facial being the commonest.^1^,^5^,^6^ Due to subtle course of the abdominal actinomycosis and non-specific radiological findings, it is difficult to diagnose preoperatively.^5^,^7^ In most instances, it mimics malignancy.^1^,^2^,^5^ We present here a case of an elderly diabetic with no known risk factors diagnosed with abdominal actinomycosis following surgery.

## CASE REPORT

A 61-year-old diabetic female patient, presented with the complaint of right sided abdominal swelling for the preceding two months. She had no history of trauma, surgery or immunosuppression. On examination, she had stable vitals and had an abdominal swelling. Ultrasonography showed a hypo-echoic mass arising from abdominal wall invaginating into peritoneal cavity. The case was further investigated using CT scan of abdomen and pelvis. A large soft tissue enhancing lesion, measuring 8.0 x 2.4 x 8.8 cm was noted to be arising from the mesentery and involving right lateral abdominal wall laterally and lateral wall of ascending colon extending upto the hepatic flexure medially, along with reactive adjacent wall thickening of colon. Surrounding pericolic fat stranding and puckering of the adjacent vessels were also seen. A few subcentimeter pericolic lymph nodes and multiple subcentimeter para-ortic and mesenteric lymph nodes were also present. The liver was enlarged. Radiological differentials included sclerosing mesenteritis, fibromatosis and pseudo-tumor. A right hemicolectomy was planned. Intra-operatively a hard 10 x 10 cm mass was seen arising from ascending colon and extending anterior to external oblique muscle. Approximately 15 cm of small bowel from ileo-cecal junction was found adherent to tumor. Small bowel, cecum, ascending colon and the ascending colon mass were resected. The specimen was sent for histopathology. The small bowel, cecum, appendix, ascending colon and hepatic flexure mass were received in formalin. Grossly, the small bowel mesentery along with peri-colonic fat of cecum and ascending colon showed an ulcerated nodular lesion measuring 10 x 9 x 7 cm. The lesion was adherent to wall of ascending colon and terminal ileum. At the periphery of lesion skeletal muscle fibers of external oblique muscle were seen.

Sections examined reveal focally ulcerated small bowel mucosa with its wall and mesentery exhibiting a fibrotic area showing dense lymphoplasmacytic and neutrophilic infiltrate along with granulation tissue formation. There were centrally placed colonies of Actinomycetes composed of basophilic radial filaments, highlighted on special stain Gomori Methenamine Silver (GMS) stain Periodic acid–Schiff (PAS). Features were compatible with Actinomycosis.

## DISCUSSION

Abdominal actinomycosis has been reported to be increasing (accounting for 20% of the disease amongst all its entities) while the overall incidence of the disease is known to be decreasing.^1^,^2^ The disease is difficult to diagnose and due to its subacute presentation and indolent course, maybe confused with conditions like malignancy, appendicitis, diverticulitis, inflammatory bowel disease, tuberculosis and pelvic inflammatory disease.^1^-^3^,^5^,^6^ The causes may vary but involve breach in mucosa, which may be due to perforated bowel, endoscopic procedures, trauma, appendectomy, intestinal necrosis, foreign body or due to unknown cause as is in our case.^2^,^3^ Commonly diseased sites are the ileocecal junction, transverse colon and the cecum with the appendix.^1^,^3^ It is a chronic disease with varying clinical presentations, from abscess formation and draining sinuses to granulation tissue and dense fibrosis. Sulfur granules can be observed in about 50% of actinomycosis cases, though they may be in other infections such as those caused by *Nocardia* and *Streptomyces* species.^4^,^5^

**Fig.1:**
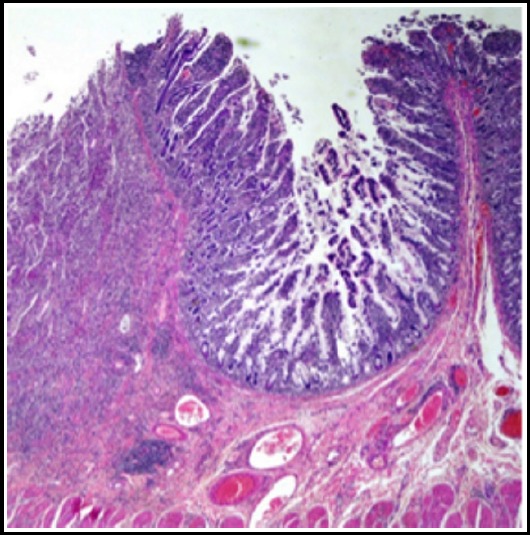
Section shows focally ulcerated small bowel mucosa. H&E 2x.

**Fig.2:**
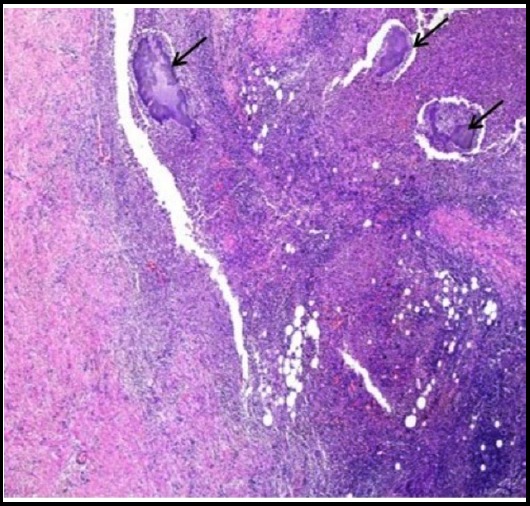
Section shows small bowel wall exhibiting dense inflammation with colonies of Actinomycetes (marked by arrow). H&E 2x.

The findings in our case are consistent with other reports with mucosal breach present as a risk factor, however uptil now no association of diabetes mellitus and abdominal actinomycosis have been found.^1^,^8^-^10^ Association of structural changes and delayed wound healing in patients with poor glycemic control, maybe the cause, however the exact mechanism is unknown.^10^ Further studies are required to reveal the actual association of abdominal actinomycosis with diabetes mellitus.

## CONCLUSION

In conclusion, abdominal actinomycosis is a rare entity with non-specific radiological findings and may mimic malignancy and should be considered as a differential in cases presenting with abdominal masses on radiology. Early intervention and antibiotic course may prevent progress of the disease and development of complications associated with the disease. Association of abdominal actinomycosis. As such further studies are needed to explore their association.

### Authors’ Contribution

**RAM:** Took the history, handled the specimen and identified the etiology, drafting the initial case report manuscript, literature search.

**YS:** Drafting the case report, literature search and gave intellectual input in the manuscript development.

**NY:** Involved in micro-photography, final drafting and review of manuscript.
